# Cell-Specific Response of NSIP- and IPF-Derived Fibroblasts to the Modification of the Elasticity, Biological Properties, and 3D Architecture of the Substrate

**DOI:** 10.3390/ijms232314714

**Published:** 2022-11-25

**Authors:** Natalia Janiszewska, Barbara Orzechowska, Kamil Awsiuk, Jakub Rysz, Svitlana Tymetska, Joanna Raczkowska

**Affiliations:** 1 The Marian Smoluchowski Institute of Physics, Jagiellonian University, Łojasiewicza 11, 30-428 Kraków, Poland; 2 Institute of Nuclear Physics Polish Academy of Sciences, Radzikowskiego 152, 31-342 Kraków, Poland; 3 Jagiellonian Center of Biomedical Imaging, Jagiellonian University, Łojasiewicza 11, 30-348 Kraków, Poland

**Keywords:** idiopathic pulmonary fibrosis IPF, nonspecific interstitial pneumonia NSIP, fibroblasts, substrate elasticity, ECM proteins, 3D pattern

## Abstract

The fibrotic fibroblasts derived from idiopathic pulmonary fibrosis (IPF) and nonspecific interstitial pneumonia (NSIP) are surrounded by specific environments, characterized by increased stiffness, aberrant extracellular matrix (ECM) composition, and altered lung architecture. The presented research was aimed at investigating the effect of biological, physical, and topographical modification of the substrate on the properties of IPF- and NSIP-derived fibroblasts, and searching for the parameters enabling their identification. Soft and stiff polydimethylsiloxane (PDMS) was chosen for the basic substrates, the properties of which were subsequently tuned. To obtain the biological modification of the substrates, they were covered with ECM proteins, laminin, fibronectin, and collagen. The substrates that mimicked the 3D structure of the lungs were prepared using two approaches, resulting in porous structures that resemble natural lung architecture and honeycomb patterns, typical of IPF tissue. The growth of cells on soft and stiff PDMS covered with proteins, traced using fluorescence microscopy, confirmed an altered behavior of healthy and IPF- and NSIP-derived fibroblasts in response to the modified substrate properties, enabling their identification. In turn, differences in the mechanical properties of healthy and fibrotic fibroblasts, determined using atomic force microscopy working in force spectroscopy mode, as well as their growth on 3D-patterned substrates were not sufficient to discriminate between cell lines.

## 1. Introduction

Among the wide variety of lung diseases that have accompanied us for years, there are still ones whose etiology is not fully understood and, even worse, which cannot be reliably diagnosed due to the lack of effective tools. The most prominent example of such diseases are idiopathic pulmonary fibrosis (IPF) and nonspecific interstitial pneumonia (NSIP), belonging to the family of interstitial lung diseases (ILDs). These two entities strongly mimic each other, they have similar clinical manifestations and overlapping histopathological and pathological images [1,2,3]; thus, the diagnostic process is long, complicated, and not always accurate, rendering proper treatment and strongly decreasing the prognosis for patients with IPF, which is a fatal disease [1,4,5,6,7,8,9,10,11,12], in contrast to NSIP, which can be easily cured [1,5,13,14,15]. Therefore, systematic research is needed on the various aspects of the fibrotic process in both diseases, to find a fast and unequivocal way to distinguish them from each other [16,17,18]. 

Tissue fibrosis, observed for NSIP and IPF results of an abnormal response to injury, is related to the overproduction of specific components of the extracellular matrix (ECM) [19,20,21,22,23], affecting normal lung functions, such as ventilation and gas exchange, with subsequent breathlessness and, finally, respiratory failure, leading to death [24,25,26]. The ECM consists of fibrous proteins, such as collagen and elastin, and structural or adhesive proteins, such as fibronectin and laminin [20,23,27,28,29,30,31]. In fibrotic diseases, collagen biosynthesis and deposition is highly up-regulated and, in fact, collagens are recognized for their hallmark [25,26,32,33,34,35,36,37,38,39]. Moreover, fibronectin, which facilitates cell migration and differentiation during the wound healing process, is present in abnormally large amounts in the areas of active fibrogenesis [36,37,39]. In addition, laminin has also been found to contribute to the development of pulmonary fibrosis [23,40,41,42,43]. Although the matrix composition of NSIP and IPF is very similar, a detailed comparison of the lung proteome of NSIP and IPF reveals some differentially regulated proteins [44]. Furthermore, research by Parra et al. [45], reports the important role of collagen in vascular remodeling, which differs for the responses to injury that occur in NSIP and IPF.

In turn, disorders in the biochemical composition of the lungs, especially pathological accumulation of collagen and cholesterol [26,28,45,46] lead to increased tissue stiffness and influence the contractile and proliferative functions of lung fibroblasts, which can result in the transformation of fibroblasts into myofibroblasts [24,27,36,39,47,48,49,50,51,52,53]. The impact of matrix elasticity on the development of IPF has already been examined [48,52,54,55], though analogous research for NSIP-derived cells is very rare [56]. Changes in the cytoskeleton organization observed for IPF and NSIP-derived fibroblasts result in differences in their mechanical properties [57], which leads to modifications of their stiffness in response to altered substrate elasticity [56]. 

Recent studies performed by Parker et al. [58] point to a possible positive profibrotic feedback loop, where initial tissue damage affects surrounding fibroblasts, causing further modification of both ECM composition and tissue stiffness, eventually leading to the spread of fibrosis [21,58,59]. Therefore, the simultaneous impact of ECM proteins and environmental elasticity on lung fibroblasts should be carefully analyzed to provide additional information on the mechanisms of the fibrotic process, possible ways to inhibit the progression of fibrosis, as well as methods to identify IPF and NSIP, the two diseases that remain hard to distinguish.

On the other hand, the macroscopic image of the fibrotic lung tissue in IPF is characterized by the presence of honeycomb lesions, which are abnormal dilated airspaces with walls composed of fibrotic tissue [60], rarely observed for other ILDs [13,16,17,18,28,61,62]. The impact of different nano- and microscale structural and topographical cues on cellular behavior is widely considered, mainly when developing materials for applications such as medical implants, cell culture systems, scaffolds for tissue engineering, or ‘organs-on-chip’ devices [63,64,65,66,67,68,69,70], showing a great variety of cellular responses, depending on the specific topographic pattern [63,71,72,73,74,75,76,77,78]. Similarly, to in vitro conditions, the topography of the cell environment within organs and tissue may influence their functions, such as adhesion, differentiation, and migration [67,71,72,78]. Therefore, the fibroblasts derived from different ILDs could potentially respond differently to patterns resembling the normal and fibrotic lung tissue, thus providing another possibility to discriminate between the diseases.

In the present paper, we examined the impact of simultaneous modification of the mechanical and biological properties of the substrate, which is typical for the fibrotin process in lung tissue, on the properties of IPF- and NSIP-derived fibroblasts. Modification of mechanical properties was performed by using two polydimethylsiloxane (PDMS) substrates with different elasticities, namely ‘soft’ PDMS with Young’s modulus E equal to 600 kPa and ‘stiff’ PDMS with E = 1.5 MPa. In turn, the biological properties of the substrate were changed by covering PDMS with the ECM proteins collagen, laminin, and fibronectin, whose aberrant abundance is characteristic of fibrotic diseases. In turn, substrates that mimic the 3D structure of the lungs were prepared using two approaches. The first was based on the method described by Yuen et al. [79], where the standard PDMS was admixed with sugar particles to obtain a porous structure. In the second, PDMS substrates were prepared by molding the SU-8 master with random and honeycomb patterns, designed using a photoplotter. The resulting patterns were recorded using optical microscopy and a profilometer. The growth and morphology of cells were traced using fluorescence microscopy. In addition, for substrates of different elasticity modified with collagen, the stiffness of cells was examined using atomic force microscopy (AFM) working in force-spectroscopy (FS) mode. The performed analysis of the results aimed to find parameters that would enable the identification of each examined cell line, and propose a novel approach for the ILD diagnostic process.

## 2. Results

**Figure 1:**
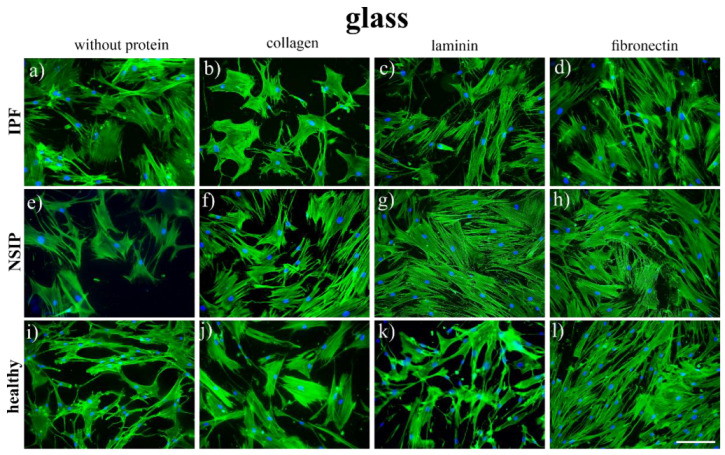
Fluorescence micrographs of the actin cytoskeleton (green) and nuclei (blue), recorded for IPF- and NSIP-derived, as well as healthy fibroblasts cultured on control glass substrates (**a**,**e**,**i**) covered with collagen (**b**,**f**,**j**), laminin (**c**,**g**,**k**), and fibronectin (**d**,**h**,**l**). The scale bar corresponds to 100 μm (objective magnification 20×).

To determine the impact of ECM proteins on IPF- and NSIP-derived fibroblasts, control glass substrates were covered with collagen, laminin, and fibronectin. After 72 h of culture, the fibroblasts were fixed and fluorescently stained to visualize the actin cytoskeleton (green) and nuclei (blue). Representative fluorescence images are presented in Figure 1.

The fluorescence images of the cells cultured on the control glass sample show that healthy cells have elongated shapes, typical for fibroblasts (Figure 1i), whereas both fibrotic cell lines are rather polygonal, with an increased cell area, which is characteristic of myofibroblasts. The modification of glass with laminin (Figure 1c,g,k) does not lead to any significant changes, the shapes of the cells are similar to those of uncoated glass, and their number changes slightly only for NSIP-derived fibroblasts. In turn, for glass coated with fibronectin, a significant increase in cell number is observed for all cell lines, but their shapes are preserved. In contrast, for glass modified with collagen, the number of cells remains almost the same as for the control glass sample, but the morphology of healthy fibroblasts becomes significantly affected (Figure 1j) and they transform from elongated to a more polygonal form, with increased cell area.

**Figure 2:**
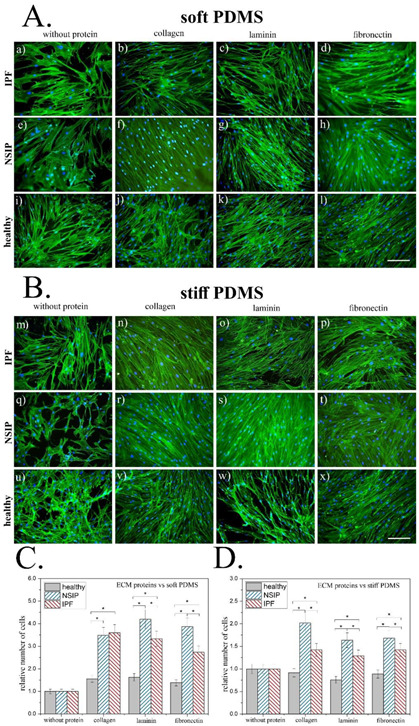
IPF- and NSIP-derived, as well as healthy fibroblasts cultured on two types of PDMS substrates with different treatments. (**A**,**B**) Fluorescence micrographs of the actin cytoskeleton (green) and nuclei (blue), recorded for IPF- (**a**–**d**,**m**–**p**) and NSIP-derived (**e**–**h**,**q**–**t**), as well as healthy fibroblasts (**i**–**l**,**u**–**x**) cultured on (**A**) soft and (**B**) stiff PDMS substrates covered with collagen, laminin, and fibronectin. The scale bar corresponds to 200 μm (objective magnification 10×). (**C**,**D**) Relative number of healthy, IPF- and NSIP-derived fibroblasts cultured on (**C**) soft and (**D**) stiff PDMS substrates covered with collagen, laminin, and fibronectin. Statistical significance marked with * was achieved for *p* < 0.01; error bars indicate the SD (*n* ≥ 20).

To examine the collective impact of mechanical and biological modification of the substrate on healthy and IPF- and NSIP-derived fibroblasts, cells were cultured for 72 h on soft (E = 600 kPa) and stiff (E = 1.5 MPa) PDMS substrates, covered with three ECM proteins. After culture, cells were stained and visualized using fluorescence microscopy, as presented in Figure 2A for soft PDMS and in Figure 2B for stiff PDMS modified with ECM proteins.

The most obvious impact of the modification of soft PDMS that is visible in the recorded micrographs is the significant increase in the number of cells on substrates decorated with proteins, compared to the uncoated PDMS (Figure 2A). This effect is mostly pronounced for both fibrotic cell lines and slightly less effective for healthy cells and does not show any noticeable dependence on the used proteins. Moreover, the characteristic polygonal morphologies of IPF- and NSIP-derived fibroblasts are visible only for the uncoated soft PDMS and cannot be observed for the substrate covered with proteins. In addition, the fibroblasts–myofibroblasts transformation, postulated for healthy fibroblasts cultured on glass coated with collagen, is not visible here. 

Similarly, for stiff PDMS (Figure 2B) the number of fibrotic cells is significantly higher on proteins-coated substrates, while the number of healthy fibroblasts remains almost unchanged. Although both IPF- and NSIP-cells present rather elongated shapes, their area is noticeably larger, compared to the cells cultured on soft PDMS. In turn, healthy fibroblasts preserve their characteristic elongated shape for all proteins used for substrate modification, and no morphological transition to myofibroblasts may be observed.

The impact of the modification of soft and stiff PDMS with ECM proteins on the growth of healthy and fibrotic fibroblasts was quantitatively analyzed. For this issue, the relative number of cells, defined as the ratio between the number of cells enumerated on a substrate modified with a given ECM protein and their number on the ‘pure’ PDMS, was calculated for soft (Figure 2C) and stiff (Figure 2D) PDMS. 

For soft PDMS (Figure 2C) the number of healthy cells increases by approximately 50% for substrates covered with proteins. In turn, the number of IPF-derived fibroblasts cultured on soft PDMS modified with collagen and laminin is 3.5 times higher compared to ‘bare’ PDMS, and more than 2.5 times higher for cells cultured on soft PDMS covered with fibronectin. On the other hand, the number of NSIP-derived fibroblasts for collagen-covered soft PDMS is almost the same as that for IPF-ones. However, for substrates covered with laminin and fibronectin, it is noticeably higher, achieving 400% growth, compared to the number of cells cultured on the uncoated soft PDMS.

For stiff PDMS (Figure 2D), the number of healthy fibroblasts is reduced on the protein-covered substrates, indicating a slightly adverse effect of increased matrix stiffness accompanied by the presence of ECM proteins towards healthy cells. In turn, the number of fibrotic fibroblasts is higher, but the observed increase is much lower compared to the soft PDMS. For IPF-derived fibroblasts, the number of cells increases by approximately 50% for all examined proteins, whereas the number of NSIP-derived cells is the highest and almost doubles for collagen-covered stiff PDMS, and increases by approximately 75% for substrates decorated with laminin and fibronectin.

**Figure 3:**
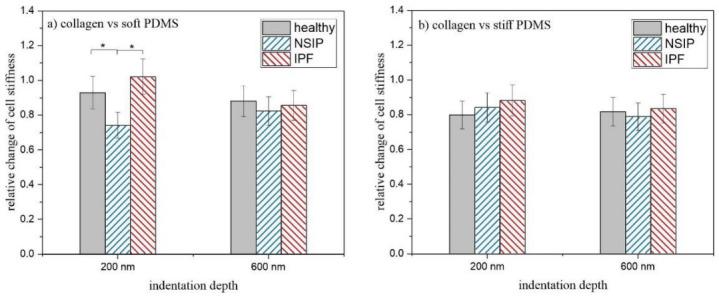
Relative change of cell stiffness calculated for healthy, IPF- and NSIP-derived fibroblasts cultured on (**a**) soft and (**b**) stiff PDMS substrates covered with collagen. Statistical significance marked with * was achieved for *p* < 0.01; error bars indicate the SD (*n* ≥ 100).

Further, the stiffness of the fibroblasts was determined using AFM-based force spectroscopy for indentation depths equal to 200 and 600 nm, providing information from different cell areas [80,81,82]. Measurements were made for cells cultured on collagen-covered soft and stiff PDMS substrates, and compared with the mechanical properties of cells cultured on uncoated PDMS [56]. The relative change in cellular stiffness, determined for the soft and stiff PDMS substrates, is presented in Figure 3a,b, respectively.

The results, presented in Figure 3, show that, for all cell lines, the coverage of stiff PDMS with collagen leads examined to a reduction of 20% in cell stiffness for both indentation depths compared to fibroblasts cultured on the uncoated substrate (Figure 3b). In turn, for fibroblasts cultured on soft PDMS modified with collagen (Figure 3a), the impact of protein on cell stiffness depends on the indentation depth. For deep cellular regions, no differences between cell lines are observed and the Young’s modulus is reduced by ~20%, similar as for stiff PDMS. However, for the measurements performed for shallower cellular regions, significant differences in mechanical properties of healthy, IPF- and NSIP-derived fibroblasts may be reported. The stiffness of IPF-derived fibroblasts is the same as for the uncoated substrate, while it is reduced for other cells, by ~10% for healthy cells and by ~30% for NSIP-derived ones.

To determine the impact of substrate topography on the growth of healthy and fibrotic fibroblasts, they were cultured on 3D regular and isotropic patterns. For this purpose, 3D PDMS substrates were prepared using two approaches. In the first, porous structures were fabricated in PDMS material by incorporating sugar (normal or fine) or salt particles into the PDMS mixture, which were removed after solidification of the substrate.

**Figure 4:**
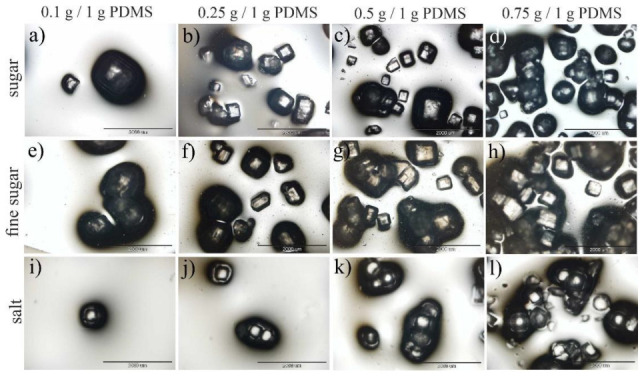
Three-dimensional PDMS structures prepared by (**a**–**d**) adding sugar, (**e**–**h**) fine sugar, and (**i**–**l**) salt to the PDMS mixture. The scale bar corresponds to 2000 μm.

**Figure 5:**
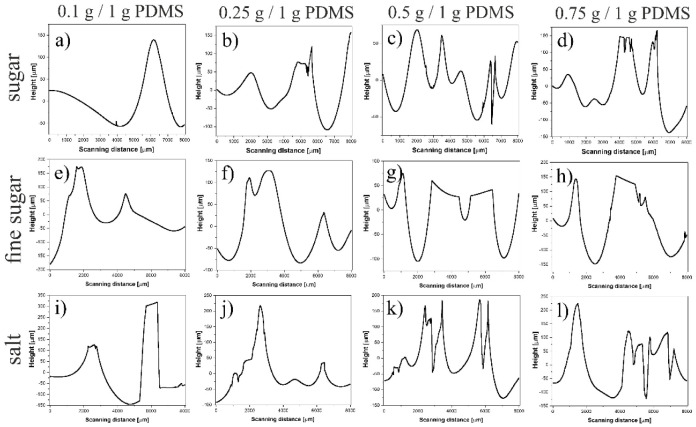
Cross-sectional profiles of 3D PDMS structures prepared by adding (**a**–**d**) sugar, (**e**–**h**) fine sugar, and (**i**–**l**) salt to the PDMS mixture.

This method, although very simple, did not enable the production of structures mimicking a sponge-like lung architecture (Figure 4). Despite the type of particles used, owing to their small amounts, they were randomly distributed but very rare (Figure 4a,e,i), whereas for higher amounts of additives, they tended to aggregate instead, forming homogeneous patterns (Figure 4d,h,l). After dissolution of the particles, 3D porous structures, similar to those presented in Figure 4, were obtained, with the depth of the pores, determined using a profilometer, in the range of 50–150 μm (Figure 5). 

**Figure 6:**
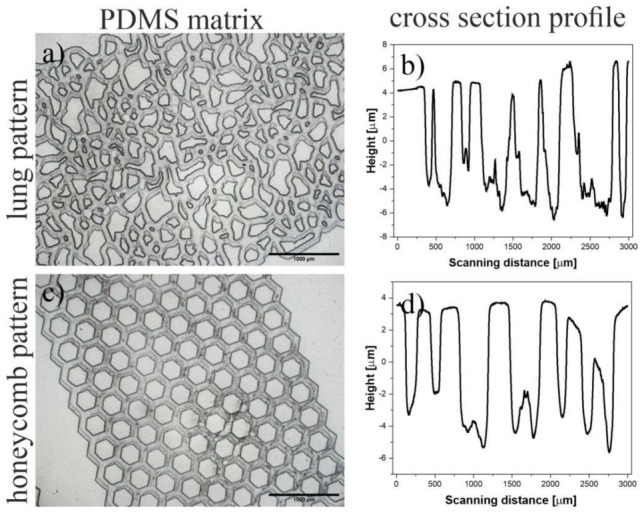
(**a**,**b**) Isotropic and (**c**,**d**) honeycomb masters used to fabricate 3D structures by PDMS molding.

In the second approach, topographical patterns were prepared using soft lithographic molding of PDMS on SU-8 masters, fabricated using foil masks printed on a home-built photoplotter. As a result, 3D PDMS structures mimicking natural (Figure 6a) and, typical for IPF, honeycomb (Figure 6c) lung architectures were fabricated. The depth of the pores, determined using a profilometer, was equal to 14.01(32) and 10.65(34) μm for normal and honeycomb patterns, respectively.

**Figure 7:**
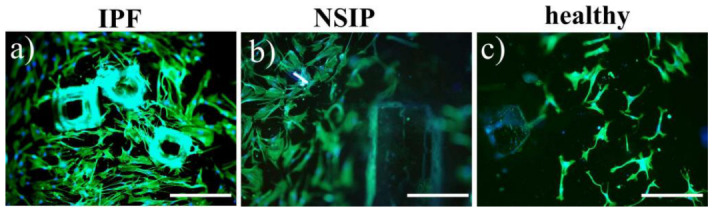
Fluorescence images of (**a**) IPF-, (**b**) NSIP-derived, and (**c**) healthy fibroblasts cultured on 3D PDMS structures prepared by adding sugar. The scale bar corresponds to 200 μm (objective magnification 10×).

**Figure 8:**
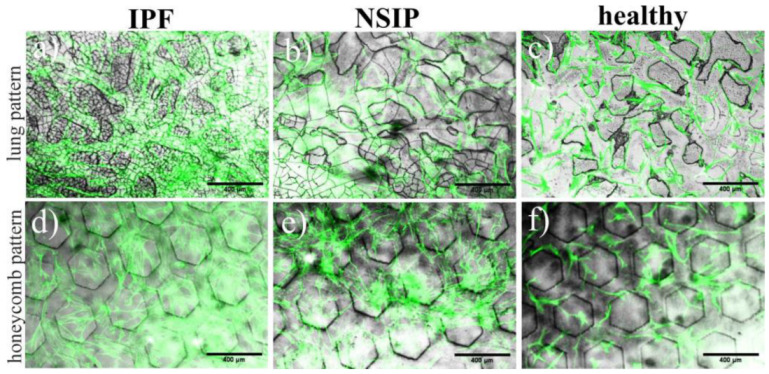
Fluorescent images of (**a**,**d**) IPF-, (**b**,**e**) NSIP-derived, and (**c**,**f**) healthy fibroblasts cultured on 3D PDMS structures mimicking (**a**–**c**) natural and (**d**–**f**) honeycomb lung architecture prepared by molding and recorded using optical microscopy. The scale bar corresponds to 200 μm (objective magnification 10×).

The produced PDMS structures were used as substrates for the culture of IPF- and NSIP-derived, as well as healthy fibroblasts. Figure 7 and Figure 8 present the growth of cells cultured on isotropic (Figure 7 and Figure 8a–c) and regular (Figure 8d–f) PDMS patterns, traced using fluorescence microscopy.

The comparison of fluorescence micrographs shows that the number of fibrotic cells cultured on isotropic substrates prepared using the first method (Figure 7a,b) is significantly larger compared to healthy ones (Figure 7c). In addition, a tendency to adhere preferentially to pattern ridges can be observed for IPF-derived fibroblasts.

Representative images resulting from a combination of fluorescence micrographs, visualizing the growth of all examined cell lines, and optical images, illustrating the PDMS patterns prepared using the second method, are presented in Figure 8. The number of healthy fibroblasts is noticeably smaller compared to the fibrotic cells, with no tendency to reproduce the substrate pattern. In turn, the number of IPF- and NSIP-derived fibroblasts is comparable for both types of substrates. For the honeycomb pattern, neither of these two cell lines form structures that mirror the original motif of the substrate (Figure 8d,e). In contrast, for 3D PDMS that resembles the natural lung architecture, the arrangement of both cell lines echoes the substrate pattern (Figure 8a,b). Furthermore, this effect is more evident for IPF-derived fibroblasts (Figure 8a).

## 3. Discussion

To find and enhance any potential difference between IPF- and NSIP-derived fibroblasts, which could enable their identification on the cellular level, the impact of three parameters which are mostly relevant for these entities, i.e., substrate elasticity, architecture and modification with the key ECM proteins, was considered. 

The biochemical properties of lung tissue are strongly affected by the proceeding fibrotic process. The abnormal response to lung tissue injury leads to disorders in the composition of ECM, mainly overproduction of collagen and fibronectin, as well as aberrant abundance of laminin, which affects lung fibroblasts [25,26,34,35,36,37,40]. To study this effect and search for the potential differences in cellular response to substrate modification between examined cell lines, especially between fibrotic cells derived from IPF and NSIP, fibroblasts were cultured on glass substrates covered with three ECM proteins and fluorescently labeled to visualize their actin cytoskeleton and nuclei. The fluorescence images of the cells cultured on the control glass sample show that although the number of cells is similar for all examined cell line, their morphology differs significantly, and both elongated shapes, typical of healthy fibroblasts, and polygonal forms, characteristic of myofibroblasts, responsible for an abnormal production of collagen [83,84,85], may be observed. The coverage of glass with laminin and fibronectin results in an increase in the number of fibroblasts, whereas for glass coated with collagen, the number of cells remains almost the same as for the control glass sample, but the morphology of healthy fibroblasts transforms from elongated into a more polygonal form, with increased cell area. Such change may be associated with the phenotypic transformation from fibroblasts into myofibroblasts, caused by the collagen, similar as for lung tissue, where pathological accumulation of collagen is considered to be the main reason of fibroblasts—myofibroblasts transformation [26,28,45,46]. However, to verify this hypothesis, fibroblasts should be additionally stained to visualize αSMA, the protein which has been found to be upregulated in lung fibrosis and cancer [86,87]. The comparison of the morphology of fibroblasts visualized on uncoated glass enables the discrimination between heathy and fibrotic cells, but the IPF- and NSIP-derived fibroblasts cannot be distinguished. In addition, a more advanced, comparative analysis of the modification of the morphology of fibroblasts cultured on collagen and their counterparts cultured on uncoated PDMS differentiates only healthy cells. 

Disorders in ECM composition, especially the high abundance of collagen, may also affect the mechanical properties of the tissue, leading to its stiffening. Although the impact of tissue elasticity on lung fibroblasts has already been studied [48,52,53,54], the effect of simultaneous modification of mechanical and biological properties of the environment, which, in fact, is more suitable for the fibrotic process in lung tissue, has not been examined extensively. This issue deserves attention, since the interactions with biologically modified substrates of different elasticities should differ for the examined cell lines, providing a potential tool to discriminate between them. To verify this hypothesis, soft (E = 600 kPa) and stiff (E = 1.5 MPa) PDMS substrates were modified using the ECM proteins collagen, laminin, and fibronectin, the aberrant abundance of which is characteristic of the fibrotic process. Successful decoration of the PDMS with proteins was confirmed using ToF-SIMS. Then, modified PDMS was used as substrates for culturing of IPF- and NSIP-derived, as well as healthy lung fibroblasts. The impact of simultaneous change of substrate elasticity and its biological properties on cells was monitored using fluorescence microscopy for soft (Figure 2A) stiff (Figure 2B) PDMS, and analyzed quantitatively in terms of the relative change in cell number after coverage of the PDMS substrate with proteins (Figure 2C,D).

The results obtained for soft PDMS show enhanced growth of all lung fibroblasts, which is significantly more effective for fibrotic cells, thus promoting the increase in their number and leading to the progression of fibrosis. A similar effect was observed for stiff PDMS; however, here the increase in the number of fibrotic cells was significantly weaker compared to the soft substrate. Moreover, a slightly adverse effect of increased stiffness of the matrix accompanied by the presence of ECM proteins toward healthy cells was observed for this substrate. The fibroblasts–myofibroblasts transformation, postulated for healthy fibroblasts cultured on glass coated with collagen is not visible here, neither for soft and stiff PDMS, nor for that coated with ECM proteins. 

In addition to the specific interactions between ECM proteins and lung fibroblasts, which promote cell growth, the coverage of PDMS with proteins also changes its wetting properties. Uncoated PDMS is highly hydrophobic, with a water contact angle equal to ~108 deg. [77,78], while most mammalian cells prefer to adhere to and grow on surfaces that are moderately hydrophilic, i.e., with contact angles between 40 deg. and 70 deg. [70,88,89], which are close to the values reported for proteins [90,91,92,93]. Therefore, the observed increase in the number of cells on substrates decorated with proteins may be related also to modified nonspecific hydrophobic/hydrophilic interactions. 

To analyze the combined effect of modified elasticity and biological properties of the substrate on healthy, IPF- and NSIP-derived fibroblasts quantitatively, their relative number, defined as the ratio between the number of cells visible on the protein-covered substrate and their number on the uncoated substrate, was calculated for soft and stiff substrates. Although the general conclusions of numerical analysis are similar to the observations made for fluorescence micrographs, which showed an increase in cell number for both PDMS substrates covered with proteins, here, significant differences between cells cultured on soft and stiff PDMS may be concluded. For soft PDMS, the presence of ECM proteins improves the growth of all lung fibroblasts but this process is significantly more effective for fibrotic cells, promoting their increase and leading to the progress of fibrosis. In turn, the impact of ECM proteins on fibrotic cells for a stiffer substrate is significantly weaker than for a softer one.

As shown in Figure 2C,D, the elasticity- and cell-dependent response of fibroblasts to substrate modification with ECM proteins results in well-defined differences in the relative numbers calculated for healthy, IPF- and NSIP-derived fibroblasts, thus providing an excellent tool for discrimination between them. For the stiff PDMS substrate, each of the examined cell lines may be precisely identified on the basis of the analysis of the impact of PDMS coverage with ECM proteins on the number of cells. In the case of soft PDMS, similar identification may be performed for substrates covered with laminin and fibronectin, whereas for soft PDMS coated with collagen, only healthy fibroblasts may be distinguished, and no difference is observed for the fibrotic ones.

The fibrotic process that occurs in the progression of ILDs is related to the reorganization of the cytoskeleton architecture and results in altered mechanical properties of disordered cells compared to healthy ones, as well as in different responses to the altered stiffness of the substrate [56]. However, the presence of ECM proteins, especially collagen, can additionally influence the properties and functions of lung fibroblasts, modifying also their interactions with substrates of different elasticities. To verify this issue, the stiffness of healthy, IPF- and NSIP-derived fibroblasts was measured for cells cultured on soft and stiff PDMS covered with collagen. Cells are complex objects, with nuclei and various organelles characterized by different mechanical properties; therefore, measurements were performed for two indentation depths, equal to 200 and 600 nm, providing information from different cell areas [80,81,82]. The measured E ranges from ~ 12 to 19 kPa, depending on the type of cell and experimental conditions, and is close to the physiological range of the Young’s modulus of pulmonary tissue (~2–10 kPa) [52,54,94,95]. However, to determine the impact of collagen modification on the mechanical properties of lung fibroblasts, the relative change of stiffness of cells cultured on modified PDMS seems to be more informative than its absolute value. The ratio between the Young’s modulus determined for cells cultured on collagen-covered PDMS and its value obtained for cells cultured on uncoated PDMS revealed that the mechanical properties of IPF- and NSIP-derived fibroblasts change differently only for cells cultured on soft PDMS. The differences, observed for outer regions of cells, indicate that the impact of matrix elasticity and abundance on mechanical properties of lung fibroblasts should not be considered independently. 

The substrate topography is known to affect cultured cells [63,71,72,73,74,75,76,77,78]. On the nanoscopic scale, the subcellular mechanisms are mainly affected, while on the microscopic scale, the topography of the substrate impacts the whole cell behaviors, such as cell morphology [88,89,96]. For lung fibrosis, the homogeneous porous architecture of normal lung tissue changes due to the fibrotic process into a honeycomb pattern [53,62,97,98]; therefore, the topography on the microscopic scale could also be a factor that impacts the behavior of fibroblasts derived from IPF and NSIP differently. To study this issue, 3D PDMS substrates were prepared using two approaches, based on the addition of soluble particles to the PDMS mixture or their molding with a predefined master, and used as substrates for the culture of IPF- and NSIP-derived fibroblasts, as well as healthy fibroblasts. For the isotropic patterns prepared using the first method, the growth of fibrotic cells was significantly more effective. In addition, a tendency to adhere preferentially to pattern ridges can be observed for IPF-derived fibroblasts, suggesting that they could be more sensitive to topographical changes in the environment. However, this effect may also be related to the characteristic dimensions and spatial distribution of pores, which differ significantly between the substrates prepared using this method. Therefore, another method that provides reproducible substrates was proposed to produce 3D PDMS patterns that resemble the natural and honeycomb lung architecture, characteristic of fibrotic tissues. 

The analysis of cell growth on these substrates supports the observations made for cells cultured on substrates prepared with sugar particles. In addition, the number of healthy fibroblasts here is noticeably smaller compared to the fibrotic cells. Moreover, for 3D PDMS that resembles the natural lung architecture, the arrangement of both fibrotic cell lines echoes the substrate pattern, significantly more effectively for fibroblasts derived from IPF, suggesting that they are the most sensitive to topographical changes of the environment. This effect may be related to the natural tendency of IPF-derived fibroblasts to spontaneously form spatially organized patterns, arising from local interactions between fibroblasts that generate fibrosis, which influence tissue stiffness in a positive feedback loop [99]. However, observed differences in cellular response to substrate patterns are insufficient to enable discrimination between healthy and IPF- and NSIP-derived fibroblasts.

## 4. Materials and Methods

### 4.1. Preparation of PDMS Substrates

The PDMS mixture was prepared using commercially available Sylgard 184 (Dow Corning, Midland, MI, USA) with a base-to-curing agent mass ratio of 10:1. Next, it was admixed with benzophenone (Sigma-Aldrich, Darmstadt, Germany) dissolved in xylene (200 mg/mL, POCH Gliwice, Poland), at a mass ratio of 1:100, and degassed. The PDMS substrates were prepared on a 25 mm round coverslip glass using a spin-coating technique (KW-4A, Chemat Technology, Los Angeles, CA, USA), resulting in an elastomeric film with a thickness of ~60 µm. The spinning speed was set to 500 rpm. To obtain soft substrates, a fraction of substrates was exposed to UV light (400 W mercury lamp, providing uniform surface irradiation with a wavelength of 254 nm) for 5 h. Finally, the samples were baked for 15 min at 150 °C. 

### 4.2. Modification with ECM Proteins

Three proteins were used to modify glass and PDMS substrates: collagen (BD Biosciences, Franklin Lakes, NJ, USA, catalog number: 354245), laminin (MP Biomedicals, Irvine, CA, USA, catalog number: 114956-81-9) and fibronectin (Sigma-Aldrich, Darmstadt, Germany, catalog number: F0895). First, the solutions of proteins in phosphate buffered saline (PBS, Sigma-Aldrich, Darmstadt, Germany) were prepared with a concentration equal to 125 μg/mL. The substrates were then completely covered with protein solution for 15 min, thoroughly rinsed with PBS and distilled water to remove non-attached proteins, and dried under a N_2_ stream. The successful modification of PDMS substrates with proteins was verified using time-of-flight secondary ion mass spectrometry, which confirmed the presence of CN^−^ and CNO^−^ ions, characteristic of proteins and not observed for PDMS, on the modified substrates.

### 4.3. 3D Patterning

To obtain 3D patterns in PDMS substrates, two methods were used. In the first, sugar or salt was added to the PDMS mixture with to give 0.1:1, 0.25:1, 0.5:1, and 0.75:1 weight ratios. The substrates were then prepared as described in Section 4.1 and additionally placed in an ultrasonic bath containing distilled water, to remove sugar or salt particles. In the second method, SU-8 masters were used to prepare 3D PDMS substrates that mimic natural and honeycomb lung architectures. The masters were fabricated in a standard photolithographic process using foil masks printed on a home-built photoplotter. First, the glass slides were cleaned by sonication in acetone and isopropanol, then a permanent negative epoxy photoresist was spin coated on top (SU-8 50), forming layers of 2–15 m thickness. The samples were then soft-baked on a hot plate for 120 sec at 65 °C and exposed to a mercury lamp through masks with various patterns. In the final step, photoresist was again annealed on a hot plate (post-bake) and developed in an SU-8 developer. The SU-8 master was then used as a template to prepare PDMS substrates.

### 4.4. Profilometry

To examine the 3D structures on PDMS substrates, their profiles were recorded using a Dektak XT (Bruker, Bremen, Germany) profilometer, equipped with a 12.5 µm radius stylus. For each sample, at least six profiles were collected in the standard hills and valleys module, to determine the lateral and vertical dimensions of the 3D structure.

### 4.5. Cell Culture

Normal human lung fibroblasts (NHLF) were purchased from Lonza (Basel, Switzerland, catalog number CC-2512), IPF-derived fibroblasts (LL97A) were purchased from ATCC (Manassas, VA, USA catalog number ATCC-CCL-191), and NSIP-derived fibroblasts (MN) were obtained from the patient sample, using standard protocol [82]. For the experiments, cells from passages 4–6 were used. All cell lines were cultured in the fibroblast-dedicated culture system containing the base medium FBM TM (Lonza, Basel, Switzerland, catalog number CC-3131) and the FGM TM SingleQuots TM supplements (Lonza, Basel, Switzerland, catalog number CC-4126) in culture flasks, in a CO_2_ incubator that provides 95% air/5% CO_2_ atmosphere. PDMS substrates and glass were placed in a Petri dish and sterilized for one hour under UVC light (germicidal lamp, λ = 254 nm) in a laminar flow chamber (Nu425, NuAire, Plymounth, MN, USA). After sterilization, a solution with cells (cell density 100,000/cm^2^) was placed on the surface of the sample. The Petri dish was then kept in the CO_2_ incubator for 72 h. To prove the reproducibility of the results, the experiments were repeated at least three times for each cell line. For each experimental sequence, two or three identical samples were prepared and measured.

### 4.6. Fluorescence Microscopy

For fluorescent staining of the actin fibers of cells and the cell nucleus, the following protocol was applied. First, cells were fixed to the substrate by immersion in a solution of 3.7% paraformaldehyde (Fluka, Sigma-Aldrich, Darmstadt, Germany) for 15 min at room temperature. Subsequently, a 0.2% Triton X-100 solution at 4 °C was added for 4 min, followed by washing the samples with PBS buffer for 2 min. To dye the actin cytoskeleton, cells were incubated with a solution of Alexa Fluor 488 conjugated with phalloidin (Alexa Fluor 488 Phalloidin, Thermo Fisher Scientific, Geel, Belgium, catalog number: A12379) in 1.5 U dilution for 40 min and then thoroughly washed (3 times for 2 min with PBS buffer). Subsequently, cells were incubated with a 1 µg/mL solution of Hoechst 34580 dye (Thermo Fisher Scientific Geel, Belgium, catalog number: H21486) for 9 min to stain the cell nuclei, then washed again (3 times for 2 min with PBS buffer). For each cell line (NHLF, LL97A, and MN), the fluorescent images were collected using an Olympus IX51 microscope equipped with a 100 W mercury light source (Olympus, Hamburg, Germany, U-LH100HG), a U-MWIG2 filter (λexit = 530–550 nm, λemit = 590 nm) and a U-MNB2 filter (λexit = 470–490 nm, λemit = 520 nm). Fluorescent images were recorded using an XC30 digital camera (Olympus) equipped with CellSense Dimensions 1.14 software (Olympus, Hamburg, Germany). For each experimental run, ten fluorescent images from two or three substrates with stained cells were collected. Then, the cells were counted using home-developed software [100], providing straightforward information about the number of cell nuclei. Statistical significance between the number of cells cultured on different substrates was achieved for *p* < 0.01.

### 4.7. Force Spectroscopy

Force spectroscopy measurements and the collection of elasticity maps from cells were carried using an atomic force microscope (AFM, model XE-120, Park System, Suwon, Korea) with commercially available silicon nitride AFM probes (ORC8-10 D, Bruker, Bremen, Germany). The samples covered with a monolayer of cells were placed inside a Petri dish lid, filled with cell culture medium (FGM–2 Fibroblast Growth Medium-2 BulletKit, Lonza, Basel, Switzerland, catalog number: CC-3132), and mounted on the AFM. The tip then approached close to the cell surface and force curves were collected within a grid of 5 × 5 points from about 40 different cells, for each substrate and time point. To estimate the quantity of the relative Young’s modulus, only an approach part of the collected force curve was analyzed using the Hertz contact model, with a paraboloid approximation of the probing tip shape [101].

### 4.8. Statistical Analysis

Statistical analysis was performed by multivariate ANOVA, using Origin (OriginLab, Northampton, MA, USA). Statistical significance between groups was determined by performing Bonferroni’s post hoc analysis. Statistical significance was achieved for *p* < 0.01.

## 5. Conclusions

The present paper aimed at investigating the impact of the substrate, mainly its coverage with proteins, elasticity, and topography, on the behavior of IPF-derived, NSIP-derived, and healthy fibroblasts, as well as identifying parameters that enable precise differentiation between all cell lines, especially between both fibrotic ones. First, the biological properties of glass were modified by its decoration with three ECM proteins—collagen, laminin, and fibronectin—and used as substrates for the culture of healthy and fibrotic fibroblasts. Further, the combined effect of modified elasticity and biological properties of the substrate, mostly relevant for the fibrotic process in lung tissue, was examined for healthy, IPF- and NSIP-derived fibroblasts cultured on a soft and stiff PDMS substrate covered with ECM proteins and quantitatively analyzed by the ratio between the number of cells visible on protein-covered substrates and their number on uncoated substrates. 

In the next step, the impact of simultaneous modification of substrate elasticity and its biological properties on the mechanical properties of lung fibroblasts was examined, using AFM-based force spectroscopy. The results obtained indicate that different types of fibroblasts respond differently to the change in mechanical properties of the environment, accompanied by the presence of collagen. However, this effect is very subtle and may be observed only for the shallow regions of cells cultured on soft PDMS. 

Finally, the impact of topography on cell growth was examined for 3D PDMS substrates fabricated using two approaches. A comparison of the growth of all studied cell lines on 3D PDMS substrates revealed a significantly larger amount of fibrotic cells compared to healthy ones on all topographic patterns. Furthermore, for 3D PDMS that resembles natural lung architecture, the arrangement of fibroblasts derived from IPF and NSIP mirrors the substrate pattern. In fact, this effect is slightly more effective for IPF-derived cells, suggesting their great sensitivity to the topographical changes of the environment.

The performed experiments confirm altered interactions of healthy, IPF- and NSIP-derived fibroblasts with biologically, mechanically, and topographically modified substrates. The cell-specific response of fibroblasts to modification of the soft and stiff substrate with ECM proteins resulted in well-defined and significant differences in the relative numbers of different cells, providing an excellent tool for discrimination between fibroblasts derived from IPF and NSIP, as well as the healthy ones, and enabling a novel approach for the ILD diagnostic process. In turn, differences in mechanical properties of cells, as well as in their response to topographic patterns of the substrate, are very subtle and the proper identification of each examined cell line based on these parameters is more challenging and requires carefully adjusted experimental conditions. To enhance the impact of topography on fibrotic cells, the PDMS architecture could be additionally modified to also mimic the nanoscopic fibrous structure of the basement lung membrane, which might also affect cellular properties.

## Data Availability

Not applicable.
